# Follicular thyroid carcinoma metastasis to the facial skeleton: a systematic review

**DOI:** 10.1186/s12885-017-3199-3

**Published:** 2017-03-28

**Authors:** Varun V. Varadarajan, Elizabeth K. Pace, Vatsal Patel, Raja Sawhney, Robert J. Amdur, Peter T. Dziegielewski

**Affiliations:** 1 Department of Otolaryngology (ENT), University of Florida, Gainesville, FL 32610 USA; 2 University of Florida College of Medicine, Gainesville, FL USA; 3 Department of Pathology, University of Florida, Gainesville, FL USA; 4 University of Florida Health Cancer Center, Gainesville, FL USA; 5 Department of Radiology, University of Florida, Gainesville, FL USA

**Keywords:** Follicular thyroid carcinoma, Head and neck surgery, Endocrine surgery, Thyroid neoplasm

## Abstract

**Background:**

Follicular thyroid carcinoma (FTC) metastasis to the facial skeleton is exceedingly rare. A case of FTC metastasizing to the mandible is presented and a systematic review of the literature describing thyroid metastasis to the facial skeleton is performed.

**Case presentation:**

A 73-year-old female presented with metastatic FTC to the mandible and underwent total thyroidectomy, segmental mandibulectomy, bone impacted fibular free flap reconstruction, and adjuvant radioactive iodine treatment. The PubMed database was searched for literature describing thyroid cancer with facial skeleton metastasis using the key words “thyroid,” “cancer,” “carcinoma,” “metastasis,” and “malignancy” with “oral cavity,” “maxilla,” “mandible,” “sinus,” “paranasal,” and “orbit.” Reports that only involved the soft tissues were excluded. Systematic review revealed 59 cases of well-differentiated thyroid cancer with facial skeleton metastasis: 35 mandibular metastases (21 = FTC), 6 maxilla metastases (2 = FTC), 9 orbital metastases (4 = FTC), and 11 paranasal sinus metastases (7 = FTC). Treatment included surgery, RAI, external beam radiotherapy (XRT), or a combination of these modalities. The one, two, and five-year survival rates were 100%, 79%, and 16%, respectively.

**Conclusion:**

Facial skeleton metastasis of FTC is a rare clinical challenge. Optimal treatment appears to include total thyroidectomy and resection of involved structures with or without adjuvant treatment.

## Background

Follicular thyroid carcinoma (FTC) is the second most common thyroid carcinoma. It accounts for ~10% of thyroid malignancies, with a higher occurrence in women aged 40–60 years [1]. Follicular thyroid carcinoma is known to disseminate hematogenously and metastasize in advanced cases. Distant metastases are seen in ~10–15% cases, with bone and lungs as preferred metastatic targets [2]. FTC metastases to the facial skeleton are exceedingly rare and present a treatment challenge.

FTC facial bone metastasis can present in the gnathic bones, the paranasal sinuses, or the orbit. Metastasis to the facial skeleton may be the first clinical sign of an underlying malignancy and clinical presentation varies depending on site of presentation as well as the primary site [3–7]. Oral cavity and maxillofacial region metastasis is uncommon and represent 1–2% of all oral and maxillofacial malignancies [3–5]. Prognoses of such lesions are assumed to be poor; however, there is a paucity of evidence to guide management of these scenarios.

In this report, a case of FTC metastasizing to the mandible is presented and a systematic review of the literature is performed. The purpose is to describe the clinical presentation, treatment, and survival outcomes of thyroid metastasis to the facial skeleton.

## Case presentation

A 73-year-old female patient was evaluated at the Head and Neck Surgery Clinic at the University of Florida. Her presenting complaint was numbness and swelling of her left mandible and an intraoral lesion associated with recurrent bleeding episodes. Symptoms were present for several weeks and had initially been presumed to represent an episode of sialadenitis by an outside provider. Her past medical history was significant for a thyroid nodule and no chronic medical conditions. She had no history of tobacco or alcohol abuse. Physical exam demonstrated a left mandibular lesion approximately 5 cm in size, with fullness of the gingival mucosa overlying the mass. A mucosal punch biopsy was performed and the histology demonstrated a pyogenic granuloma.

**Fig. 1:**
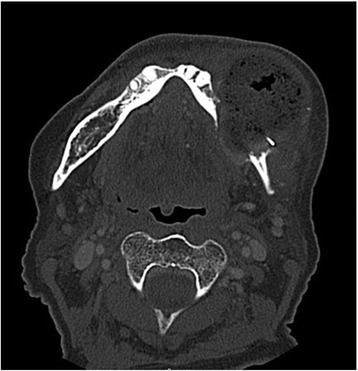
Preoperative computerized tomography depicting an aggressive mass arising from the left mandible

Computerized tomography (CT) showed an aggressive mass destroying the mandibular body (Fig. 1) as well as enlarged pulmonary nodules and a lytic bone lesion at T10. Imaging also demonstrated a multinodular thyroid gland with minimal irregularity along the anterior right border. A 4.6 cm nodule was noted in the right thyroid lobe. Fine needle aspiration of the right thyroid mass was interpreted as a follicular lesion of undetermined significance (FLUS). Because the pathology findings were inconsistent with the CT scan, an open biopsy in the OR was performed. A mucosal incision was made over the mass and a biopsy was taken. The lesion was found to be extremely friable and bled significantly requiring ligation of the facial artery. Final pathology demonstrated FTC.

**Fig. 2:**
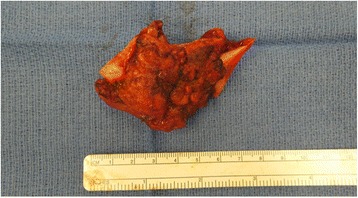
Left hemimandibulectomy specimen

**Fig. 3:**
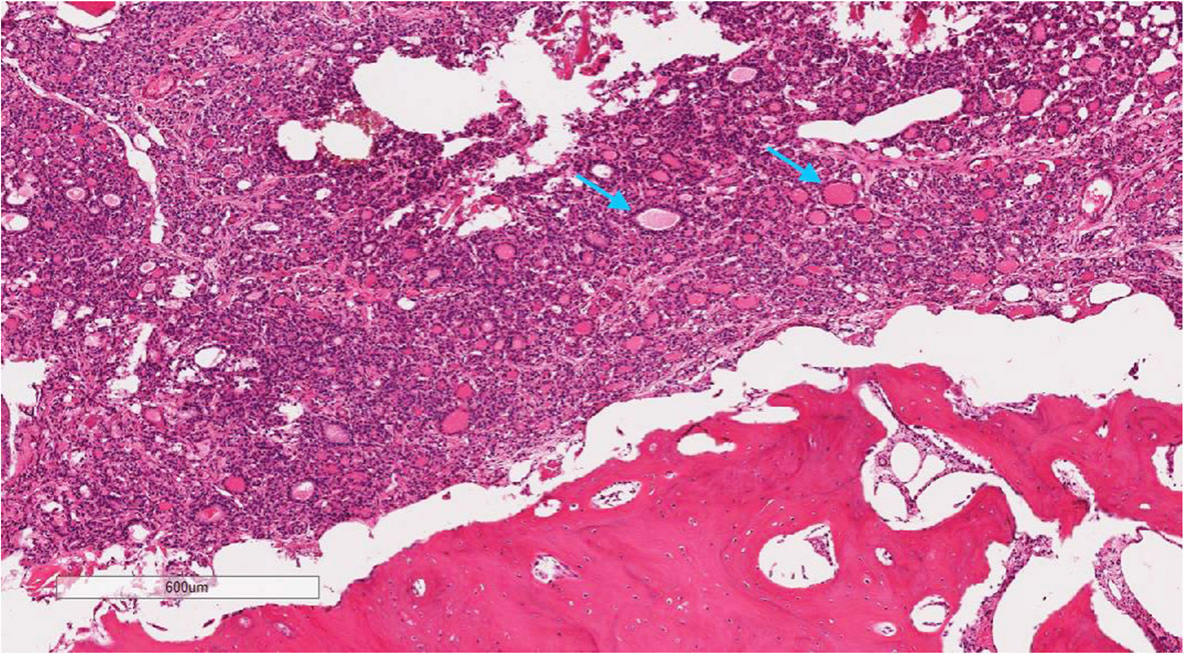
Photomicrograph of hematoxylin and eosin stain at 4X power depicting tumor cells infiltrating and destroying mandibular bone. Several tumor cells are forming follicles characteristic of follicular carcinoma of the thyroid (blue arrows)

Multidisciplinary tumor board review recommended surgery followed by radioactive iodine and external beam radiotherapy. The patient underwent total thyroidectomy, neck dissection, segmental mandibulectomy, and bone-impacted fibular free flap reconstruction [6]. Intraoperative findings included a 10 cm thyroid mass of the right thyroid lobe that extended beneath the sternum to the innominate vein. A segment of mandible was taken from left angle to right parasymphysis, resulting in a defect from right lateral incisor to angle of mandible (Fig. 2). Reconstruction was undertaken via a right bone-impacted fibular free flap with skin paddle in addition to a 2.0 mm mandibular reconstruction bar. Final pathology showed mandibular metastasis of FTC with extension into the tongue and soft tissues of the neck (Fig. 3). Margins were negative. The 4.6 cm thyroid follicular carcinoma appeared to arise from a calcified pre-existing degenerative follicular adenoma. There was evidence of capsular invasion and extensive lymphovascular invasion. The patient underwent post-operative stereotactic body radiation to the T-10 metastatic lesion and 200 mCi of radioactive Iodine-131. She has been disease free for 18 months.

A systematic review of the English literature was performed using PubMed, Medline, Embase, and Scopus databases. Search terms describing FTC presenting as a metastatic lesion in the facial skeleton were compiled and implemented. These terms included: “thyroid,” “cancer,” “thyroid carcinoma,” “thyroid cancer”, “metastasis,” and “malignancy” with “oral cavity,” “maxilla,” “mandible,” “sinus,” “paranasal,” and “orbit.” Papers were gleaned for diagnoses of well-differentiated thyroid cancer, FTC, and metastases to the facial skeleton. Reports of metastasis that only involved the soft tissues were excluded. The reports were organized by subsite: mandible, maxilla, jawbone not otherwise specified, nasal cavity or paranasal sinus, and orbit. Data points obtained from literature review included age, gender, primary oncologic diagnosis, site of metastasis, clinical presentation, treatment modality, survival outcome, and time to follow-up were obtained. Statistical analysis was performed with SPSS 23.0 software package (SPSS Inc., Chicago, IL). Survival was estimated by a Kaplan-Meier analysis to the account for censored data. Survival by treatment was analyzed and compared using the log rank test.

Literature review identified 64 studies reporting 97 cases of thyroid cancer metastasis to the facial skeleton in the English literature. All metastases were present at the time of presentation. 59 case reports specified well-differentiated thyroid cancer as the diagnosis. Table 1 demonstrates details of these cases. 38 case reports did not specify the diagnosis and were not included [7–18]. The gender distribution was 9 males, 48 females, and 2 cases in which gender was not specified. While the majority of metastases were to the mandible, other craniofacial sites were also found to be involved.

**Table 1:** List of reported cases of thyroid cancer metastasis to the facial skeleton

Author of report	Age, Gender	Primary tumor	Site of presentation	Clinical presentation	Treatment of metastases	Survival	Time to follow-up
Agarwal et al. [26]	45, F	FTC	Mandible	Facial swelling	Resection	Yes	2 weeks
Algahtani et al. [40]	66, F	FTC	Mandible	Pathologic fracture	Resection	NR	NR
Anil et al. [72]	61, F	FTC	Mandible	Mandibular swelling	NR	NR	NR
Bhadage et al. [28]	40, F	FTC	Mandible	Facial swelling	NR, referred out	NR	NR
Bingol et al. [27]	33, F	PTC	Mandible	Painless mass of mandibular angle	Surgery, RAI	No	5 years
Colella et al. [75]	50, F	PTC	Mandible	Pain and swelling in RMT	NR	NR	NR
Draper et al. [44]	NR, F	FTC	Mandible	Ulcerated oral lesion	XRT, RAI	NR	NR
Erdag et al. [23]	53, F	PTC	Mandible	Right sided facial swelling	Surgery, RAI	Yes	2.5 years
Essakalli et al. [41]	50, F	PTC	Mandible	Painful swelling of jaw	Resection, RAI	Yes	2 months
Germain et al. [42]	50, F	PTC	Mandible	Jugular, carotid lymphadenopathy	Resection	Yes	17 months
Ismail et al. [30]	70, F	FTC	Mandible	Pain, *“loose teeth”*	NR	NR	NR
Kahn and McCord [31]	82, F	FTC	Mandible	Painful oral swelling	XRT, surgical salvage	No	18
Kumar RVK et al. [32]	58, F	FTC	Mandible	Painless facial swelling	Resection, mandible reconconstructive bar, second stage thyroidectomy	Yes	2 years
Lavanya et al. [29]	76, M	FTC	Mandible	Painless mandibular swelling	NR, referred out	NR	NR
Liu et al. [33]	66, M	PTC	Mandible	Cheek mass	Resection, RAI	Yes	3 years
Markitziu et al. [46]	69, F	PTC	Mandible	Facial swelling	XRT	Yes	18 months
McDaniel et al. [76]	77, F	FTC	Mandible	Pain, swelling	Resection, parotidectomy, RAI	Yes	4 years
Meyer and Shklar [3]	51, F	FTC	Mandible	NR	NR	NR	NR
Muttagi et al.: 2 cases [34]	NR	PTC	Mandible	NR	Surgery	NR	NR
	NR	FTC	Mandible	NR	Surgery	NR	NR
Nishikawa et al. [39]	83, F	PDFTC	Mandible	Painful swelling of jaw and face	None	No	19 months
Osguthorpe and Bratton [35]	53, M	FTC	Mandible	Slowly enlarging parotid mass	Resection, RAI	Yes	3 years
Ostrosky et al. [25]	72, M	FTC	Mandible	Painful vascular lesion	Resection, iliac crest graft	NR	NR
Pasupula et al. [36]	40, F	FTC	Mandible	Painful left parotid swelling	Resection	NR	NR
Tamiolakis et al. [4]	69, F	PTC	Mandible	Facial swelling, mucosal ulcerations	Inoperable	NR	NR
Tovi et al. [47]	33, M	FTC	Mandible	Mimicking AVM	RAI	No	17 days
Vazifeh et al. [37]	58, F	FTC	Mandible	Facial swelling	Resection	NR	NR
Vishveshwaraiah et al. [38]	56, F	FTC	Mandible	Painless facial swelling, face and lip paresthesia	NR, referred out	NR	NR
Vural and Hanna [24]	64, F	FTC	Mandible	Tender, pre-auricular mass	Resection, post op iodine ablation	Yes	6 weeks
Zandi et al. [11]	64, F	FTC	Mandible	NR	NR	NR	NR
	75, F	FTC	Mandible	NR	NR	NR	NR
	63, F	PTC	Mandible	NR	NR	NR	NR
	44, F	PTC	Mandible	NR	NR	NR	NR
	35, F	PTC	Mandible	NR	NR	NR	NR
	51, F	PTC	Mandible	NR	NR	NR	NR
Antunes et al. [22]	13, F	PTC	Maxilla	NR	NR	NR	NR
Fatahzadeh et al. [48]	43, F	PTC	Maxilla	Hemorrhagic mass with ulceration and bleeding	XRT	NR	NR
Hefer et al. [52]	58, M	FTC	Maxilla	Left hard palate pain	Resection	Yes	2 years
Kumar CS et al. [51]	31, F	FTC	Maxilla	Painful swelling, mobile teeth	RAI	Yes	7 years
Nikitakis et al. [49]	63, M	PTC	Maxilla	Painful swelling of right posterior maxilla	XRT, palliative chemotherapy	Yes	2 years
Slim et al. [50]	67, F	PTC	Maxilla, zygoma	Painless malar swelling	Resection, postoperative iodine ablation	Yes	NR
Cinberg et al. [64]	80, F	FTC	Maxillary sinus	Epistaxis	RAI	NR	NR
Altinay et al. [69]	68, F	FTC	Nasal Cavity, orbit, skull base	Left eye puffiness, proptosis, vision Loss, facial numbness	XRT	Yes	1 month
Malhotra et al. [60]	55, F	FTC	Orbit (Anterolateral orbit)	Proptosis, vision Loss	Resection, RAI	NR	NR
Rocha Filho et al. [57]	66, F	PTC	Orbit (Frontal bone)	Frontal bone mass	Palliative chemo	Yes	NR
Bernstein-Lipschitz et al. [55]	56, F	FTC	Orbit (Lacrimal fossa, orbital roof)	Diplopia, ptosis, orbital Pain	Resection	NR	NR
Shyla et al. [54]	70, F	PTC	Orbit (Posterior orbit extending to ethmoid bone)	Vision loss	Resection, XRT	Yes	NR
Boughattas et al. [56]	25, F	PTC	Orbit (Supraorbital)	Asymptomatic	NR	NR	NR
Daumerie et al. [58]	59, F	PTC	Orbit (Supratemporal quadrant)	Left upper eyelid swelling, exophthalmos	RAI	Yes	2 months
Pagsisihan et al. [59]	49, F	PTC	Orbit (Supraorbital)	Supraorbital mass	RAI	Yes	6 months
Argibay-Vasquez [77]	53, F	PTC	Sphenoid	Headache, paresthesia in the right eye region, left monocular diplopia	RAI, subtototal resection, XRT, RAI	Yes	3 years
Yamasoba et al. [67]	34, F	FTC	Ethmoid, sphenoid, maxillary, intracranial	Cheek hypoesthesia, hearing Loss	Embolization, resection	Yes	NR
Renner et al. [65]	61, F	FTC	Sphenoid sinus	Epistaxis, anosmia, visual loss	RAI, XRT	Yes	5 months
Barrs et al. [63]	54, F	FTC	Sphenoid sinus, orbit	Visual loss	RAI, XRT	No	5 years
Altman et al. [68]	81, M	FTC	Sphenoid, ethmoid, skull base	Headache	XRT	No	1 year
Freeman et al. [78]	50, M	PTC	Sphenoid, ethmoid	Facial pain, proptosis of the left globe, left horner’s syndrome	XRT, RAI	Yes	1 year
Madronio et al. [79]	53, F	PTC	Sphenoid, ethmoid	Headache, galactorrhea, vision loss	Surgical debulking	Yes	13 months
Cumberworth et al. [66]	74, F	FTC	Sphenoid, frontal, ethmoid, and maxillary sinuses	Nasal obstruction	None	No	1 week after diagnosis

*FTC* Follicular Thyroid Carcinoma, *NR* not reported, *PTC* Papillary Thyroid Carcinoma, *RAI* Radioactive Iodine Therapy, *XRT* External Beam Radiation Therapy

Treatment varied between studies and included: Surgery with or without preoperative embolization and radioactive iodine therapy, external beam radiation (primary or adjuvant treatment), and palliative chemotherapy. 22 patients were treated with surgery as initial treatment with or without postoperative radioactive iodine or external beam radiation. 11 patients were treated with external beam radiation as primary treatment. 14 reports did not specify treatment. 4 patients were treated with palliative care; 2 of these patients received palliative chemotherapy. Cases were grouped into: a surgical arm (those treated with surgery and RAI) and a non-surgical arm. 32 studies reported survival outcome and 27 studies reported time-to-follow up. 24 patients survived treatment and 8 patients expired.

Overall survival for all patients at 2 years was 96% and at 5 years was 59%. Disease specific survival at 2 years was 96% and at 5 years was 72%. Patients treated with surgery and RAI versus those treated by non-surgical means were compared. There was no statistical difference in overall survival (*p* = 0.27) with the surgical group having 2 and 5 year overall survival of 100% and 71%, respectively and those in the non-surgical arm having rates of 92% and 46%.

**Fig. 4:**
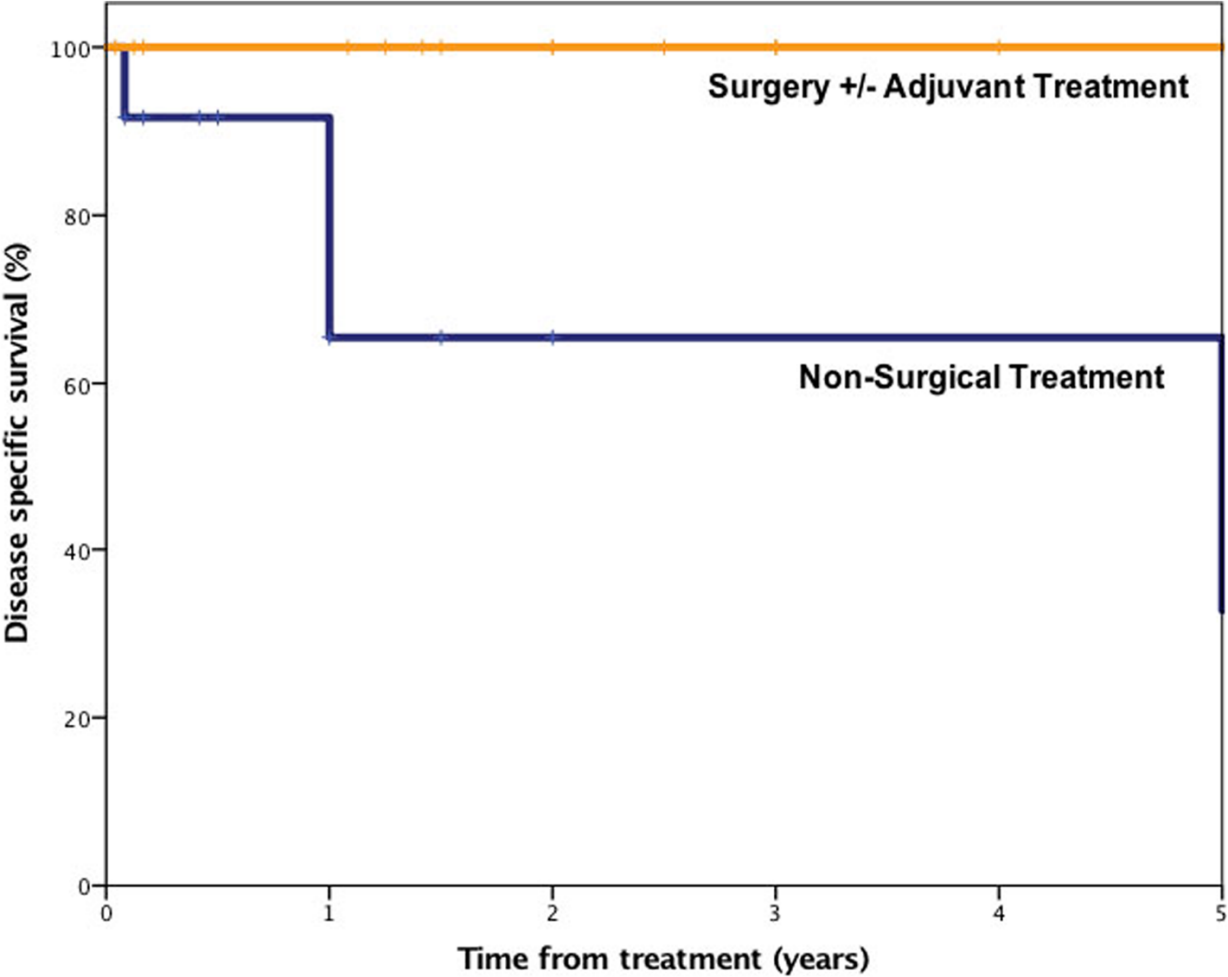
Disease specific survival for patients with well-differentiated thyroid cancer metastatic to the facial skeleton treated with surgery as initial treatment with adjuvant radioactive iodine therapy versus non-surgical treatment (external beam radiation)

Disease specific survival for all patients at 2 years was 96% and at 5 years was 72% (Fig. 4). There was a statistically significant difference in disease specific survival (DSS) between patients treated with surgery and RAI versus those treated by non-surgical means (*p* = 0.03). DSS for surgically treated patients at 2 and 5 years was 100% and for non-surgically treated patients was 92% and 46%, respectively.

## Discussion and conclusions

FTC is the second most common thyroid cancer, following papillary thyroid carcinoma (PTC). 10–15% of FTCs will disseminate hematogenously via angioinvasion. The most common sites of metastases include bone and lungs and less commonly brain, liver, bladder, and skin. Bone metastases can occur in the vertebral bodies followed by the pelvis, femur, skull, and ribs [2, 19]. Treatment often involves high dose radioiodine; however, bony metastases are less likely to concentrate radioiodine, and thus, the efficacy is estimated at 55%. External beam radiation therapy may be used for palliation [2].

Metastasis comprises 1% of all oral-maxillofacial malignancies. Primary sites of tumors metastatic to the facial skeleton are most commonly from the breast and lungs [20]. Thyroid malignancy represents 2% of facial skeleton metastasis [20] and 4.2–6.1% of all jaw metastases [7, 15, 21] 41% of facial skeleton metastasis from thyroid cancer occurs in the mandible; 59% of these metastases are well-differentiated thyroid cancer. There have been 41 reported cases in the literature of thyroid malignancy with metastasis to the mandible of which 21 reported cases were FTC [4, 9–12, 22–47]. There have been 6 reported cases of metastasis to the maxilla; 2 were FTC [22, 48–52].

The majority of metastatic tumors to the mandible present with facial swelling and an osteolytic lesion. A rapid progression of intraoral or extraoral swelling associated with chin paresthesia and pain is not uncommon [21, 29, 36, 53]. As the tumor invades oral mucosa, a granulation-like mass may form and result in significant bleeding, infection, fractures, and disturbances in swallowing and mastication [32, 40].

Isolated facial skeleton metastasis may be treated with surgical resection, radioactive iodine, external beam radiation or combinations of the three. The patient presented here was treated with a composite resection and radioactive iodine. Her defect was reconstructed with a bone-impacted osteocutaneous fibula free flap. Follow-up CT scanning demonstrated that the neo-mandible retained a dense bone stock from the bone impaction. Free flap reconstruction for metastatic thyroid cancer to the mandible has only been reported once in the literature [42]. The current case is the first report of a bone-impacted fibular free flap used in this scenario.

Metastatic thyroid carcinomas are also reported in the orbit and paranasal sinuses. 9 cases have been described in the bony orbit; 4 of these were FTC [16, 54–61]. Surgical debulking of the metastatic foci may restore vision in cases of sudden onset vision loss; radioiodine treatment has also been documented as treatment for tumors that uptake iodine. External beam radiation can also be an option. There are 17 reported cases of thyroid malignancy presenting as a paranasal sinus mass (14.1%); 7 of these cases were FTC [8, 13, 14, 17, 18, 62–69]. Two cases presented simultaneously in the paranasal sinus and the bony orbit [63, 69]. Clinical manifestations include epistaxis, nasal obstruction, visual disturbances, and facial or intraoral swelling [70, 71]. The maxillary sinus is the most commonly involved sinus followed by the sphenoid sinus, ethmoid, and frontal sinus [70, 72]. The vertebral venous plexus, which allows retrograde spread of tumor emboli, could explain the etiology of paranasal sinus and orbital metastasis [71, 73]. Craniofacial resection or debulking with or without preoperative vascular embolization can be considered, however, the proximity of the metastatic tumor to the skull base may preclude surgical extirpation [59, 67, 69]. Radioiodine therapy, external beam radiation, chemotherapy, or palliative therapy can be considered in these patients [57, 59, 68, 74].

Survival analysis suggests that surgical resection of involved craniofacial structures with or without adjuvant treatment is the optimal treatment for FTC metastatic to the facial bones. Given the rarity of the condition, the sample size is limited; however, survival analysis demonstrated convincing statistically significant advantages with surgical resection. Treatment plans should be formulated with a multidisciplinary team involving surgical oncology, radiology, pathology, endocrinology, medical oncology, radiation oncology, and possibly palliative care.

In conclusion, facial skeleton metastasis of FTC is a rare clinical challenge. If feasible, surgical-based treatment options offer the best survival outcomes. When mandibular defects are present, reconstruction with a bone impacted fibular free flap may provide a reconstruction with a robust bone stock.

## Abbreviations

CT: Computerized tomography
FTC: Follicular thyroid carcinoma
NR: Not reported
PTC: Papillary thyroid carcinoma
RAI: Radioactive iodine therapy
XRT: External beam radiation therapy
